# Inequities in human papillomavirus vaccination among children aged 9–14 years old under constrained vaccine supply in China

**DOI:** 10.1186/s12939-024-02199-z

**Published:** 2024-05-31

**Authors:** Xiaomin Wang, Jiayi Pan, Bo Yan, Ran Zhang, Tianchi Yang, Xudong Zhou

**Affiliations:** 1 School of Public Health, Hangzhou Normal University, No. 2318 Yuhangtang Road, Yuhang District, Hangzhou, 311121 China; 2 Institute of Social Medicine, School of Medicine, Zhejiang University, 866 Yuhangtang Road, Xihu District, Hangzhou, 310058 China; 3 Department of Health Promotion, Education, and Behavior, Arnold School of Public Health, University of South Carolina, Columbia, SC 29208 USA; 4 Institute of Immunization and Prevention, Ningbo Municipal Center for Disease Control and Prevention, No.1166, Fan Jiangan Road, Haishu District, Ningbo City, 315000 Zhejiang Province China; 5 The Second Affiliated Hospital, School of Medicine, Zhejiang University, 88 Jiefang Road, Hangzhou, 310009 China

**Keywords:** Human papillomavirus vaccine, Health inequity, Vaccine supply, China, Cervical cancer prevention

## Abstract

**Background:**

Inequities in access to human papillomavirus (HPV) vaccine are becoming a growing critical issue globally. Few studies investigate the factors determining HPV vaccine uptake disparities when vaccine supply is constrained, especially in low- and middle-income countries. The aim of this study was to investigate inequities of HPV vaccination and related factors under the constrained vaccine supply in China.

**Methods:**

A cross-sectional survey was conducted in a developed eastern coastal province and a developing western one in China between November and December 2022. Employing multistage stratified cluster random sampling, the study collected data from parents of children aged 9–14. Mixed-effects logistic regression models with school units as random effects were used for analysis.

**Results:**

From 4,127 eligible parents (as vaccine decision makers for girls), 1,346 (32.6%) intended to vaccinate their daughters against HPV, of which 836 (62.1%) attempted to schedule a vaccination appointment. Only 16.4% succeeded in booking an appointment. More than half of the intended parents expected the imported 9-valent HPV vaccine. There were significant disparities in HPV vaccine awareness, intention, and vaccination behavior across educational, income, geographic, ethnic, gender, and health literacy levels. Vaccine awareness and intentions were higher among parents with higher socioeconomic status; however, girls from lower socioeconomic families were more likely to receive the HPV vaccine and had a higher domestically produced vaccination rate. Significant disparities exist in vaccination intentions and actual vaccination behaviors, primarily due to large supply constraints of the HPV vaccine.

**Conclusions:**

Sustained health education campaigns are needed to raise awareness of the HPV vaccine, improve health literacy, and decrease over-preference for the 9-valent HPV vaccine. A mother’s HPV vaccination behavior was positively associated with increased intention and actual vaccination behavior for her daughter. This study advocates for complementary cervical cancer prevention programs targeting both mothers and daughters.

**Supplementary Information:**

The online version contains supplementary material available at 10.1186/s12939-024-02199-z.

## Introduction

Vaccination is one of the most effective strategies for controlling, eliminating, and ultimately eradicating infectious diseases. It has significantly reduced healthcare costs, providing health, social, and economic benefits and helping to narrow the global inequity gap caused by economic differences between high-income and low- and middle-income countries (LMICs) [1]. Paradoxically, this progress has been contrasted by emerging inequities in vaccination coverage, especially with newly introduced vaccines, which have become more pronounced between countries [2, 3]. LMICs face a disproportionate burden of cervical cancer incidence and mortality [4, 5], but the distribution of the Human Papillomavirus (HPV) vaccine has been predominantly administered to girls in high-income countries (HICs) [6], often neglecting marginalized racial and socioeconomic groups within these nations, such as minorities, females, the illiterate, and the poor [7, 8]. For example, in the United States, urban adolescents have higher rates of HPV vaccination than rural counterparts [9].

The efficacy of the HPV vaccine in preventing cervical precancers among adolescent girls is well-established [10]. The World Health Organization’s (WHO) Global Strategy to Accelerate the Elimination of Cervical Cancer has set targets: by 2030, 90% of girls should receive the full course of the HPV vaccine by the age of 15 [11]. Although vaccination rates have reached 90% in some LMICs with support from international non-governmental organizations (e.g., GAVI, the Vaccine Alliance), in counties without such support, the vaccination rate is as low as 3.5% [12]. Furthermore, this situation is exacerbated by a global shortage of the HPV vaccine, coinciding with its increasing incorporation into national immunization programs (NIPs) by HICs [13, 14]. This shortage may deteriorate the inequities in HPV vaccination access [15], but few studies investigated the factors determining vaccination disparities under the condition of constrained supply in LMICs.

China accounts for 18% of the global cervical cancer burden but the Chinese NIP has not yet covered the HPV vaccine [16]. Despite the availability of domestically produced, cost-effective 2-valent HPV (2vHPV) vaccines, Chinese parents tend to prefer imported vaccines, particularly the higher-valency options [17]. However, the limited global supply of imported 4-valent HPV (4vHPV) and the 9-valent HPV (9vHPV) vaccines, coupled with restrictions on batch release in China, has led to a system known as “Vaccine Lottery” where the likelihood of securing a vaccine is only 1.7% [18]. Although a high intention to vaccinate is reported among parents of primary school children (83.3%) [19], actual vaccination rates for HPV remain low, with only 7.5% of girls aged 12–19 being vaccinated by 2020 [17].

Most of studies exhibit the equity barriers by comparing vaccinated and unvaccinated, vaccine acceptance and vaccine hesitancy groups [20–22]. However, miss attribution of equity barriers can lead to inappropriately tailored solutions. For example, studies on vaccine hesitancy often propose strategies aimed at reducing hesitancy within racial and ethnic minority groups, rather than addressing systemic issues within the public health system to enhance vaccine accessibility and affordability [23]. Another study found that caregiver’s higher awareness of HPV vaccine was associated with a higher vaccination rate [24]. Moreover, few measured the inequity factors from the whole process of vaccine awareness, intention, and behavior perspective [25]. Given the documented impact of factors such as residency, race, ethnicity, gender, wealth, and health literacy on vaccination [26], this study aims to take China as an example in the contest of constrained vaccine supply, to investigate the inequity of HPV vaccination and the related factors thus to develop a subnational level strategy to improve vaccine coverage within the country.

## Methods

### Study design and setting

We conducted a cross-sectional survey in two provinces in China between November and December 2022. The province selected were Zhejiang, a developed eastern coastal province with the fourth-highest per capita gross domestic product (GDP), and Guizhou, a developing western province ranked 22nd out of 32 participating mainland provinces in terms of GDP, to represent geographic and economic variation. A questionnaire was developed based on existing literature on HPV vaccination awareness, intentions, behaviors, and influencing factors [27, 28]. It was refined following a pilot study consisting of face-to-face interviews with 30 parents and a quantitative sample of 786 parents. Ethical approval was obtained from Zhejiang University School of Public Health (no. ZGL202103-3). The reporting of this study conforms to the Strengthening the Reporting of Observational Studies in Epidemiology (STROBE) guidelines (Appendix S1).

### Participants and data collection

To ensure an adequate sample size for subgroup analyses, we aimed to investigate 4,009 girls’ parents to satisfy the minimum sample size (Appendix S2). Multistage stratified cluster random sampling was adopted to ensure a representative sample of Chinese parents with children aged 9 to 14. One prefecture-level city per province was randomly selected, and within each prefecture-level city, one urban district and one rural county were randomly chosen as sampling sites. At each site, two primary schools and two middle schools were randomly selected. At each school, a certain number of classes from each grade from Grade 4 to Grade 9 were randomly selected. All parents of children in these selected classes were invited to participate. To guarantee the minimum sample size, we aim to include at least 84 girls’ parents per grade in each sampled school.

**Fig. 1:**
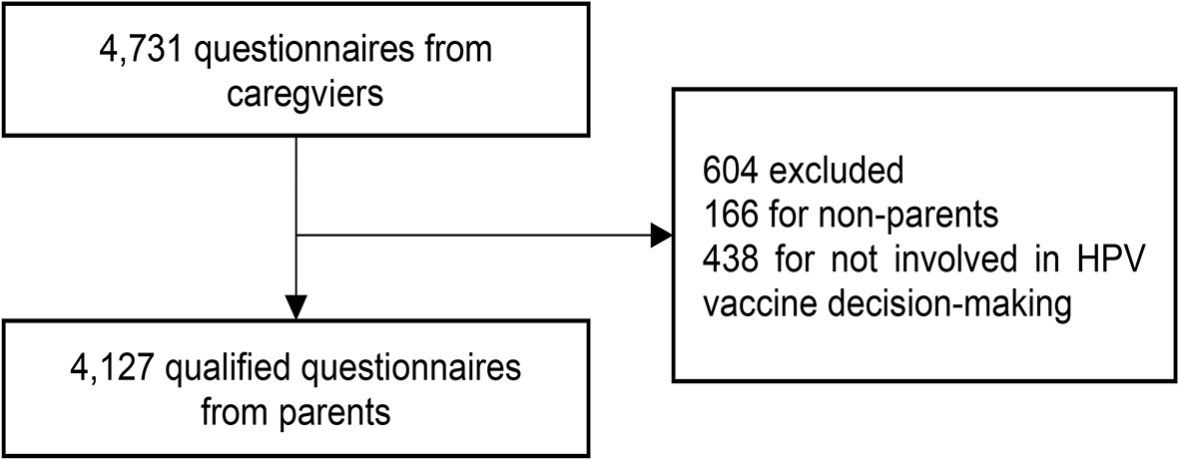
Flow chart of study participants

The questionnaire was administered to the parents responsible for making vaccination decisions for their children (Fig. 1). The first section of the questionnaire consisted of an information sheet and a consent form that was signed off by all participants. Permission to conduct the study in schools was obtained from the local Bureau of Education and the Center for Disease Control and Prevention. Participants were informed that their involvement was voluntary and that completing the questionnaire would take approximately 10 to 20 min. Teachers at each sampled class distributed the survey link to parents, and parents who agreed to participate then signed the consent form and completed the questionnaire online. The total number of girls and boys at each school was collected to calculate the response rate. A trap question was used as an attention check.

### Variables and measurement

#### Parental HPV vaccination awareness, intention, and behavior

The four dependent variables were parental awareness of the HPV vaccine, intention to vaccinate, choice of vaccine type, and actual vaccination behavior. The first three outcome variables-parental awareness of the HPV vaccine, intention to vaccinate, and choice of vaccine type were assessed by the Precaution Adoption Process Model to trace the parental decision-making process from awareness to action [29]. The response options for these three outcome variables were dichotomized as follows: “aware of the vaccine vs. not aware”, “vaccinated vs. not vaccinated”, and “intended to get vaccinated vs. not intended.” The choice of vaccine types was measured by asking intended parents “Which type of HPV vaccine do you intend to choose for your daughter?”. Responses to this question were categorized into “9vHPV vaccine” and “4vHPV/2vHPV vaccine”.

### Equity variables

#### Health literacy

The analysis incorporated questions on parental health literacy to reflect the concepts of functional health literacy, as described by Nutbeam and other scholars in this field [30–32]. Two items were included in the analysis that inquired about information access and understanding in the context of HPV vaccination: To what degree do you agree or disagree with the following statements: (1) “It is difficult for me to find correct and comprehensive information about the HPV vaccine.” (2) “It is difficult for me to understand the information I got about the HPV vaccine.” Responses were measured using a Likert scale ranging from 1 (strongly agree) to 5 (strongly disagree). These two items were combined into one continuous variable, with higher scores indicating greater health literacy.

#### Gender equity

Gender equity was assessed using three questions: (1) “Whether child’s mother got HPV vaccine uptake?”, (2) “Whether child’s mother ever received cervical cancer screening test?”, and parents’ gender. Response options for the first two questions were dichotomized into “Yes” and “No/don’t know”.

Other equity factors including girl’s ethnicity, and parents’ educational attainment, and monthly household income, were selected. Provincial development status (developed vs. developing) and residency (urban vs. rural) were selected as geographical equity variables. Girls’ date of birth (for age calculation) was a confounding variable.

### Statistical analysis

Categorical variables were described as proportions and compared using Chi-square tests. Mixed-effects logistic regression models with school units as random effects (accounting for the cluster sampling by school) were used to calculate adjusted odds ratios (OR) with 95% confidence intervals (CI). These models assess the association of various factors with parental outcomes: awareness of the HPV vaccine, intended to get the HPV vaccine, vaccinated with the HPV vaccine, and intended to choose the 9vHPV vaccine for their daughters across all groups (i.e., age, ethnicity, province, residency, parent’s educational attainment, monthly household income, parent-child relationship, HPV vaccination or cervical cancer screening service utilization by child’s mother, and parent’s health literacy). All analyses were performed with SPSS (version 22.0) and SAS software (version 9.4). No missing data were found in the analyzed data. A p-value of less than 0.05 was considered statistically significant.

## Results

The study achieved a response rate of 73.2%. Among the 4,127 girls’ parents, 78.0% were mothers, 55.6% had a monthly household income less than 5,000RMB (US$714), and 56.8% had attained an education level of middle school and below (Table 1). The mean age of the girls was 12 years, and 13.2% belonged to racial and ethnic minority (hereafter minority) groups. Participants who lived in urban and rural areas from Zhejiang and Guizhou were equally distributed.

**Table 1:** Descriptive analysis of the potential inequity determinants for HPV vaccination procedures

	Total (*n* = 4127)
Girls’ age	
9	395(9.6)
10	643(15.6)
11	671(16.3)
12	717(17.4)
13	727(17.6)
14	719(17.4)
≥15	255(6.2)
Girls’ ethnicity	
Han	3583(86.8)
Minority	544(13.2)
Province	
Zhejiang	2107(51.1)
Guizhou	2020(48.9)
Residency	
Urban	2025(49.1)
Rural	2102(50.9)
Parents’ education	
Primary and under	458(11.1)
Middle school	1886(45.7)
High school	928(22.5)
College and above	855(20.7)
Household Income (monthly)	
≤3000 (US$428)	982(23.8)
3001–5000 (US$429–714)	1313(31.8)
5001–10,000(US$715–1429)	1035(25.1)
> 10,000 (US$1429)	398(9.6)
Parent-child relationship	
Father	908(22.0)
Mother	3219(78.0)
Mother received the HPV vaccine	
No/Don’t know	3533(85.6)
Yes	594(14.4)
Mother screened for cervical cancer	
No/Don’t know	2398(58.1)
Yes	1729(41.9)
Parental health literacy score	
1 (Lowest)	1081(26.2)
2	1649(40.0)
3	355(8.6)
4 (Highest)	1042(25.2)

**Fig. 2:**
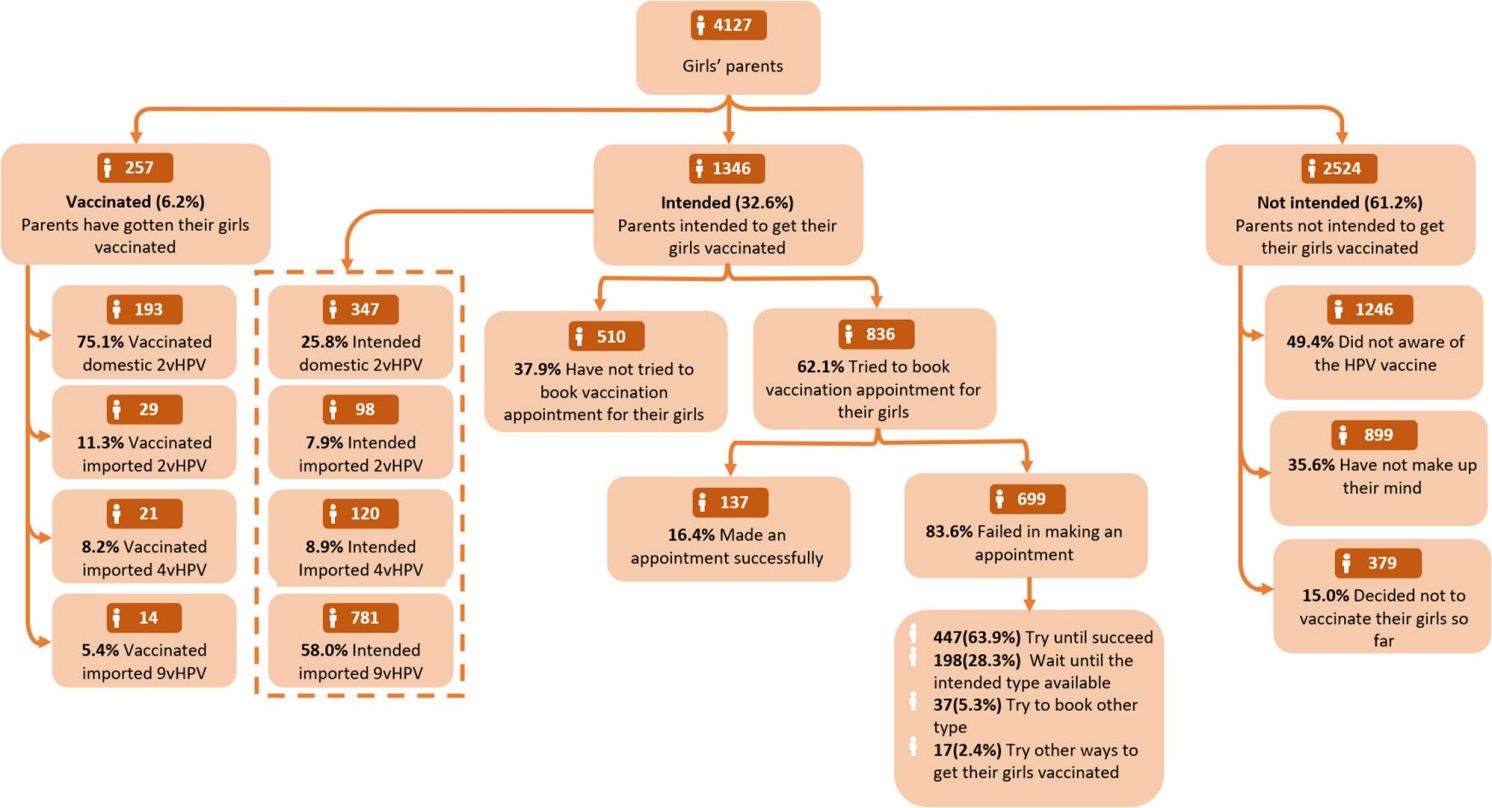
HPV vaccination awareness, intention, and behavior among girls’ parents

Figure 2 shows the decision-making process of parents regarding HPV vaccination for their daughters. Of the respondents, 32.6% (1,346) intended to get their daughters vaccinated, with 62.1% (836) of this subset attempting to book a vaccination appointment but only 16.4% of them made an appointment successfully. Among these 1,346 parents who intended to vaccinate their daughters, only a quarter preferred the domestic 2vHPV vaccine, while 58% opted for the imported 9vHPV vaccine. For those who failed in making an appointment, 63.9% stated their determination to continue trying. Among 61.2% (2,524 out of 4,127) who did not intend to get their daughters vaccinated, half were unaware of the HPV vaccine. Only 6.2% (257) of parents reported having had their daughters vaccinated, with more than three-quarters of their daughters receiving the domestic 2vHPV vaccine. Unadjusted and adjusted associations between equity factors with HPV vaccination awareness, intention, and behavior are shown in Table 2.

**Table 2:** Mixed-effects logistic regression model for inequity barriers predicting parental HPV vaccination awareness, intention, and behaviors for their girls

	Aware of the HPV vaccine *n* = 4127			Already vaccinated *n* = 4127			
	Unadjusted OR (95%CI)	Adjusted OR (95%CI)		Unadjusted OR (95%CI)	Adjusted OR (95%CI)		
Intercept	-	0.44(0.21,0.92)*		-	0.07(0.02,0.25)***		
Girls’ age	1.01(0.98,1.05)	1.07(1.02,1.13)*		1.09(1.01,1.17)*	1.03(0.94,1.13)		
Girls’ ethnicity							
Han	ref	ref		ref	ref		
Minority	0.81(0.67,0.98)*	0.97(0.79,1.19)		1.12(0.78,1.60)	0.76(0.50,1.14)		
Province							
Zhejiang	ref	ref		ref	ref		
Guizhou	0.68(0.60,0.78)***	0.78(0.62,0.99)*		2.10(1.61,2.73)***	1.79(1.18,2.71)**		
Residency							
Urban	ref	ref		ref	ref		
Rural	0.93(0.81,1.06)	0.88(0.70,1.11)		1.86(1.43,2.42)***	1.61(1.08,2.40)*		
Parents’ education							
Primary and under	ref	ref		ref	ref		
Middle school	1.11(0.89,1.38)	1.04(0.83,1.31)		0.54(0.39,0.74)***	0.64(0.45,0.92)*		
High school	1.13(0.89,1.43)	1.02(0.79,1.33)		0.28(0.19,0.43)***	0.36(0.22,0.58)***		
College and above	1.69(1.32,2.17)***	1.28(0.96,1.71)		0.14(0.08,0.25)***	0.17(0.09,0.32)***		
Household Income (monthly)							
<=3000	ref	ref		ref	ref		
3001–5000	1.19(0.99,1.41)	1.07(0.89,1.30)		0.55(0.41,0.74)***	0.86(0.61,1.20)		
5001–10,000	1.22(1.01,1.47)*	1.00(0.81,1.24)		0.42(0.29,0.59)***	0.76(0.50,1.47)		
> 10,000	1.82(1.47,2.25)***	1.22(0.95,1.58)		0.24(0.15,0.38)***	0.49(0.28,0.85)*		
Parent-child relationship							
Father	ref	ref		ref	ref		
Mother	2.00(1.72,2.34)***	1.71(1.45,2.01)***		0.57(0.43,0.75)***	0.60(0.44,0.81)**		
Mother received HPV vaccine							
No/Don’t know		ref		ref	ref		
Yes	4.09(3.13,5.34)***	3.09(2.33,4.10)***		3.89(2.97,5.10)***	7.18(5.06,10.18)***		
Mother screened for cervical cancer							
No/Don’t know	ref	ref		ref	ref		
Yes	2.16(1.87,2.49)***	1.42(1.21,1.66)***		0.75(0.57,0.97)*	0.49(0.35,0.69)***		
Parental health literacy score							
1 (Lowest)	ref	ref		ref	ref		
2	1.10(0.93,1.29)	1.17(0.99,1.38))		0.81(0.58,1.13)	0.84(0.59,1.19)		
3	2.30(1.73,3.07)***	2.07(1.54,2.78)***		0.74(0.42,1.29)	0.89(0.49,1.60)		
4 (Highest)	2.25(1.86,2.74)***	1.86(1.52,2.28)***		1.57(1.13,2.18)**	1.68(1.18,2.40)**		
	Intended to get vaccinated *n* = 4127			Intended to choose 9vHPV *n* = 1346			
	Unadjusted OR (95%CI)		Adjusted OR (95%CI)	Unadjusted OR (95%CI)		Adjusted OR (95%CI)	
Intercept	-		0.18(0.09,0.37)***	-		0.12(0.03,0.51)**	
Girls’ age	1.02(0.99,1.06)		1.06(1.01,1.12)*	0.94(0.88,0.996)*		1.05(0.95,1.16)	
Girls’ ethnicity							
Han	ref		ref	ref		ref	
Minority	0.68(0.56,0.82)***		0.78(0.63,0.96)*	0.86(0.60,1.23)		1.07(0.68,1.68)	
Province							
Zhejiang	ref		ref	ref		ref	
Guizhou	0.66(0.58,0.75)***		0.78(0.62,0.99)*	0.59(0.47,0.74)***		0.75(0.45,1.24)	
Residency							
Urban	ref		ref	ref		ref	
Rural	1.07(0.94,1.21)		1.00(0.79,1.25)	1.05(0.85,1.31)		0.85(0.52,1.40)	
Parents’ education							
Primary and under	ref		ref	ref		ref	
Middle school	1.10(0.89,1.37)		0.97(0.77,1.22)	1.37(0.88,2.13)		1.08(0.67,1.77)	
High school	1.24(0.98,1.57)		1.01(0.78,1.31)	2.29(1.44,3.65)***		1.38(0.82,2.32)	
College and above	1.62(1.28,2.05)***		1.11(0.84,1.47)	5.44(3.39,8.75)***		2.49(1.43,4.35)**	
Household Income (monthly)							
<=3000	ref		ref	ref		ref	
3001–5000	1.28(1.07,1.52)**		1.19(0.98,1.44)	1.43(1.02,2.00)*		1.03(0.71,1.51)	
5001–10,000	1.32(1.10,1.58)**		1.08(0.87,1.33)	2.85(2.00,4.05)***		1.72(1.14,2.60)*	
> 10,000	1.97(1.63,2.39)***		1.36(1.07,1.73)*	4.24(2.96,6.08)***		1.85(1.19,2.88)**	
Parent-child relationship							
Father	ref		ref	ref		ref	
Mother	1.42(1.22,1.66)***		1.16(0.98,1.37)	3.41(2.50,4.65)***		2.84(2.00,4.03)***	
Mother received HPV vaccine							
No/Don’t know	ref		ref	ref		ref	
Yes	4.98(4.11,6.03)***		3.87(3.15,4.75)***	3.04(2.29,4.03)***		2.32(1.67,3.22)***	
Mother screened for cervical cancer							
No/Don’t know	ref		ref	ref		ref	
Yes	2.42(2.13,2.75)***		1.58(1.37,1.83)***	2.99(2.39,3.75)***		1.74(1.34,2.27)***	
Parental health literacy score							
1 (Lowest)	ref		ref	ref		ref	
2	0.86(0.73,1.01)		0.90(0.76,1.07)	1.01(0.76,1.33)		0.98(0.71,1.34)	
3	1.37(1.08,1.75)*		1.21(0.94,1.57)	1.65(1.10,2.49)*		1.29(0.82,2.04)	
4 (Highest)	1.61(1.35,1.91)***		1.26(1.05,1.52)*	1.63(1.21,2.19)**		1.03(0.73,1.44)	

*p < 0.05,**p < 0.01,***p < 0.001

### Health literacy

Parents with higher health literacy showed greater awareness, intention (i.e., intention to get daughter vaccinated and intention to choose 9vHPV), and vaccination behavior (*p* < 0.05). After adjustment in the mixed-effects logistic regression model, a positive association was shown between higher health literacy and increased parental awareness of the HPV vaccine (aOR = 2.07, 95%CI [1.54, 2.78], aOR = 1.86, 95%CI [1.52, 2.28]), parental intention to get their daughters vaccinated (aOR = 1.26, 95%CI [1.05, 1.52]), and girls’ actual vaccination behavior (aOR = 1.68, 95%CI [1.18, 2.40]). However, the preference for the 9vHPV vaccine was not significantly associated with health literacy in the adjusted model.

### Gender

Mothers, acting as the primary vaccine decision-makers for their girls, exhibited a higher awareness and intention but a lower vaccination behavior for their daughters than fathers. The model indicated that mothers were more likely to be aware of the HPV vaccine (aOR = 1.71, 95%CI [1.45, 2.01]) and intended to choose the 9vHPV vaccine (aOR = 2.84, 95%CI [2.00, 4.03]). However, they were less likely to get their daughters vaccinated (aOR = 0.60, 95%CI [0.44, 0.81]).

The unadjusted model indicated that mothers who had received HPV vaccine or a cervical cancer screening test were more aware of and intended to vaccinate their daughters. Consistent with the unadjusted model, the adjusted model showed that mothers who had been vaccinated against HPV were significantly more likely to be wares of the vaccine, intended to vaccinate their daughters, choose the 9vHPV vaccine, and actually vaccinated their daughters (aOR = 3.09 [95%CI 2.33, 4.10], aOR = 3.87 [95%CI 3.15, 4.75], aOR = 2.32 [95%CI 1.67, 3.22], aOR = 7.18 [95%CI 5.06,10.18], respectively). Mothers who had received cervical cancer screening had higher awareness and intention for the HPV vaccine but lower actual vaccination rates for their daughters. The adjusted model showed that screened mothers reported higher vaccine awareness (aOR = 1.42, 95%CI [1.21, 1.66]), intention to get their daughters vaccinated (aOR = 1.58, 95%CI [1.37, 1.83]), and preference for the 9vHPV vaccine (aOR = 1.74, 95%CI [1.34, 2.27]). However, they were less likely to have their daughters vaccinated (aOR = 0.49, 95%CI [0.35, 0.69]).

### Ethnicity

Parents of minority girls reported lower HPV vaccine awareness and vaccination intentions for their daughters compared to their Han counterparts. Adjusted analyses showed that parents of minority girls were less likely to intend to get their daughters vaccinated (aOR = 0.78, 95%CI [0.63, 0.96]) than their Han counterparts.

### Education

The unadjusted model indicated that parents with college and above education had greater awareness and intention to get their daughters vaccinated, but these associations were not significant in the adjusted model. Nonetheless, parents with high school and above education attainment correlated with a greater intention to choose the 9vHPV vaccine. In addition, parents’ education attainment was negatively associated with girls’ vaccination behavior. In the adjusted model, parents with middle school, high school, and college and above education attainment showed lower odds (aOR = 0.64, 0.36, 0.17, respectively) in vaccination behavior for their daughters. Parents with college and above education level were 2.49 times more likely to intend to choose the 9vHPV vaccines for their daughters (95%CI 1.43, 4.35).

### Income

Parents with a 5,000RMB (US$714) and above monthly household income showed higher HPV vaccine awareness. Parents with lower monthly household incomes reported a higher vaccination intention but a lower vaccination behavior rate for their daughters. Consistently, in the adjusted model, parents with a monthly household income exceeding 10,000RMB (US$1,429) were more likely to intend to vaccinate (aOR = 1.36, 95%CI [1.07, 1.73]) but were less likely to actually vaccinate their daughters (aOR = 0.49, 95%CI [0.28, 0.85]).

### Geographic

Parents in Guizhou province had a higher vaccination rate than their Zhejiang counterparts (8.4% vs. 4.2%, *p* < 0.001). More parents who lived in rural areas had their daughters vaccinated, predominantly with the domestic 2vHPV vaccine (*p* < 0.01). Parents from Guizhou province had lower awareness and intention of HPV vaccine but a higher proportion of parents had their daughters vaccinated. The mixed-effects logistic regression model showed that parents from Guizhou province had a lower awareness of HPV vaccine (aOR = 0.78, 95%CI [0.62, 0.99]) but a higher HPV vaccination behavior (aOR = 1.79, 95%CI [1.18, 2.71]) for their daughters. Parents lived in rural areas were more likely to get their daughters vaccinated (aOR = 1.61, 95%CI [1.08, 2.40]).

### Age

Parents of girls with older age were more likely to aware of the HPV vaccine (aOR = 1.07, 95%CI [1.02, 1.13]) and more intended to get their daughters vaccinated (aOR = 1.06, 95%CI [1.01, 1.12]).

Sensitivity analyses supported these findings (Appendix S4).

## Discussion

To our knowledge, this is the first study to investigate the inequity of HPV vaccination awareness, intention, and behavior among girls aged 9 to 14 under the constrained vaccine supply condition. We found substantial gaps across educational, income, geographic, ethnic, gender, and health literacy level groups. Parents with higher socioeconomic status reported greater awareness and intention towards HPV vaccination. However, girls from social disadvantage families were more likely to get vaccinated, with a higher uptake of the domestic HPV vaccine. Our findings illustrate the complexity of understanding behaviors related to the HPV vaccine and provide insights for Chinese policymakers, particularly in the current situation of vaccine shortage, with goals set to increase HPV vaccination coverage among teenage girls to 90% by age 15 [33]. Findings from this study would provide strategies for other LMICs to mitigate the vaccine inequity especially when vaccine is constrained.

We found a significant gap between vaccination intention and behavior (38.8% vs. 6.2%), which may be caused by the large supply constraint of the imported HPV vaccine. Only 16.4% of parents could make an HPV vaccination appointment for their daughters successfully. Expanding vaccine manufacturing capacity in LMICs was suggested as a strategy to response to the vaccine inequity during COVID-19 pandemic [34]. However, our results discouraged the propose by indicating the high preference for imported vaccines in China. Most (74.2%) of parents intending to vaccinate their daughters would choose the imported vaccine. Moreover, among those who have tried to make a shot appointment, only 5.3% of them stated that they would try another type of vaccine. Addressing vaccine supply could lead to a 17% increase in the uptake rate, allowing the 699 respondents who were unsuccessful in securing appointments to get their daughters vaccinated. However, gaps between intentions and actions could also reflect low priority of actions, situational obstacles, initiating challenges, or difficulties in maintaining precautions that prove arduous [29]. Thus, further studies are needed to investigate the reasons for inaction among parents.

Globally, females bear almost 90% of the disease burdens preventable by the HPV vaccine [35]. Achieving high vaccination coverage among girls would improve gender equity through reducing the HPV disease burden among females [36]. Our study identified a significant mother-daughter cluster effect within families regarding cervical cancer preventive behaviors. Specifically, a mother’s HPV vaccination behavior was positively associated with awareness, intention, and actual vaccination behavior for her daughter. For example, vaccinated mothers were seven times more likely to have their daughters vaccinated. Our results echoed previous gender equity studies that higher maternal decision-making autonomy, access and control over resources positively associated with child immunization [37]. Moreover, a mother with a history of cervical cancer screening was positively associated with HPV vaccination awareness and intention for their daughters. The ongoing national cervical cancer screening free programme showed its effectiveness in raising parental awareness and intention of the HPV vaccination for their daughters [38]. However, a mother’s screening history was negatively associated with her daughter’s HPV vaccination behavior. The result was inconsistent with previous studies conducted in Belgium [39] and the Netherlands [40], which showed a 4-fold and 1.5-fold in the mother’s screening history with the daughter’s vaccination, respectively. The reason for the inconsistency might be that cervical cancer screening is free for women aged 35 to 64 in rural areas since 2009 and for all eligible women since 2019 [38, 41], while HPV vaccination costs are still out-of-pocket in China. Furthermore, the higher preference for the imported 9vHPV vaccine with mothers exhibiting a stronger preference for this option over fathers might contribute to this inconsistency. In response, we propose two programmes to benefit future cervical cancer prevention. First, a paired programme offering vaccines for daughters when their mothers register for HPV vaccination. Second, an on-site health promotion programme targeting mothers attending cervical cancer screenings to advocate for adolescent HPV vaccination.

Surprisingly, the vaccination uptake rate in Guizhou, a less developed western province, was twice that of Zhejiang, a developed eastern coastal province. Moreover, parents from rural areas and lower socioeconomic backgrounds were more likely to get their daughters vaccinated, primarily with the domestically produced 2vHPV vaccine (75.1%), despite higher socioeconomic groups’ preference for imported vaccines [19]. Because the HPV vaccine is not covered by the NIP in China, people need to pay out-of-pocket [42]. The cost for the imported 9vHPV vaccine (190.3US$/dose) is much higher than the domestic 2vHPV vaccine (46.5US$/dose) [18]. The comparatively lower price and higher accessibility of the domestically produced vaccine make it more acceptable among parents with lower socioeconomic status. On the contrary, we identified half of the intended parents would like to vaccinate their girls with imported 9vHPV. The situation of shortage will worsen when the 9vHPV expanded the age range from 16 to 26 to 9–45 years old [43]. Our study indicates that parents with the highest income and educational attainment, who experienced less gender inequity (i.e., mothers with better health service utilization), prefer the 9vHPV vaccine for their daughters. However, this preference could delay optimal vaccine timing due to the constrained supply of the 9vHPV vaccine [44]. Consequently, a continuous health education campaign is essential to improve health literacy and decrease the irrational over-preference for the 9vHPV vaccine. Moreover, our findings indicate that government-guaranteed, free, and sufficient provision of the domestically produced 2vHPV vaccine could advance health equity among girls from lower socioeconomic families. Some Chinese cities are piloting free vaccination programs for teenage girls, offering either the 2vHPV domestic or a subsidy of 600RMB (86US$) for any type [45, 46]. Consequently, our study recommends the strategy of providing the 2vHPV vaccine for free to reduce health inequity.

We found a much higher HPV vaccination rate among mothers, who are not the primary target of the vaccine [47, 48], than their daughters (14.4% vs. 6.2%). A meta-analysis also concluded that more HPV vaccines were administered to adult women than girls (5.26% vs. 2.54%) among LMICs with an HPV vaccination rate of less than 50% [12]. The current HPV vaccination rate among girls aged 9 to 14 is unsatisfactory. Health education programmes are urgently needed to raise the public awareness of primary target population for HPV vaccination are teenage girls rather than adult women.

## Limitation

This study has several limitations. First, we included only parents of school-attending girls, not enclose those who had dropped out of school. The equity barriers to HPV vaccination may be more severe among girls who were not attending school; however, this bias could be low for the dropout rate is lower than 5% in China [48]. Second, data collection relied on a self-reported questionnaire, which could introduce recall bias. Third, the study was conducted in only two out of 34 provinces in China, but we employed a multi-stage randomized sampling method to enhance representativeness across different socioeconomic and geographic areas. Fourth, we did not specify whether the girls had completed the full HPV vaccination series, potentially leading to an overestimation of the vaccination rate. Nonetheless, the WHO’s updated one-dose recommendation for girls aged 9 to 14 could mitigate this concern [47]. Finally, our study was conducted solely in China, which may raise concerns regarding external validity, as the findings may not be generalizable beyond the Chinese context.

## Conclusion

We found that one-third of parents were unaware of the HPV vaccine and identified a significant gap between vaccination intention and vaccination behavior, primarily due to supply constraints of the HPV vaccine. Although the domestic 2vHPV vaccine is available, most parents preferred the 9vHPV vaccine, which might delay the best timing for vaccinating their daughters. Continuous health education campaigns are needed to promote HPV vaccine awareness, improve health literacy, and decrease the disproportionate preference for 9vHPV vaccine. A strong mother-daughter cluster within a family in cervical cancer preventive behaviors was detected. To support cervical cancer prevention, we propose a mother-daughter paired programme offering free domestic 2vHPV vaccine for daughters and free testing for mothers, alongside health education to promote adolescent vaccination. Our findings provide evidence to tailor policies and strategies for Chinese policymakers.

### Electronic supplementary material

Below is the link to the electronic supplementary material.

**Appendix**: S1. STROBE Statement. S2. Sample size calculation. S3. Precaution Adoption Process Model modification. S4. Sensitive analysis

## Data Availability

No datasets were generated or analysed during the current study.
