# Supplementary material for: Inequities in human papillomavirus vaccination among children aged 9–14 years old under constrained vaccine supply in China
Source: Int J Equity Health. 2024 May 31;23:112. doi: 10.1186/s12939-024-02199-z (PMC11141026; doi:10.1186/s12939-024-02199-z)
Supplement: Supplementary file 1 — Appendix: S1. STROBE Statement. S2. Sample size calculation. S3. Precaution Adoption Process Model modification. S4. Sensitive analysis [file 12939_2024_2199_MOESM1_ESM.docx]

**Appendix**

**S1. STROBE Statement—Checklist of items that should be included in reports of *cross-sectional studies***

|  | Item No | Recommendation | Page No |
| --- | --- | --- | --- |
| **Title and abstract** | 1 | (*a*) Indicate the study’s design with a commonly used term in the title or the abstract | 1-2 |
|  |  | (*b*) Provide in the abstract an informative and balanced summary of what was done and what was found | 1-2 |
| Introduction | | | |
| Background/rationale | 2 | Explain the scientific background and rationale for the investigation being reported | 3-4 |
| Objectives | 3 | State specific objectives, including any prespecified hypotheses | 4 |
| Methods | | | |
| Study design | 4 | Present key elements of study design early in the paper | 4 |
| Setting | 5 | Describe the setting, locations, and relevant dates, including periods of recruitment, exposure, follow-up, and data collection | 4 |
| Participants | 6 | (*a*) Give the eligibility criteria, and the sources and methods of selection of participants | 5 |
| Variables | 7 | Clearly define all outcomes, exposures, predictors, potential confounders, and effect modifiers. Give diagnostic criteria, if applicable | 5-6 |
| Data sources/ measurement | 8* | For each variable of interest, give sources of data and details of methods of assessment (measurement). Describe comparability of assessment methods if there is more than one group | 5-7 |
| Bias | 9 | Describe any efforts to address potential sources of bias | 5,7, appendix |
| Study size | 10 | Explain how the study size was arrived at | 5 |
| Quantitative variables | 11 | Explain how quantitative variables were handled in the analyses. If applicable, describe which groupings were chosen and why | 5-7 |
| Statistical methods | 12 | (*a*) Describe all statistical methods, including those used to control for confounding | 7 |
|  |  | (*b*) Describe any methods used to examine subgroups and interactions | 7 |
|  |  | (*c*) Explain how missing data were addressed | 7 |
|  |  | (*d*) If applicable, describe analytical methods taking account of sampling strategy | 7 |
|  |  | (*e*) Describe any sensitivity analyses | 7, appendix |
| Results | | | |
| Participants | 13* | (a) Report numbers of individuals at each stage of study—eg numbers potentially eligible, examined for eligibility, confirmed eligible, included in the study, completing follow-up, and analysed | 5 |
|  |  | (b) Give reasons for non-participation at each stage | 5 |
|  |  | (c) Consider use of a flow diagram | 5 |
| Descriptive data | 14* | (a) Give characteristics of study participants (eg demographic, clinical, social) and information on exposures and potential confounders | 7 |
|  |  | (b) Indicate number of participants with missing data for each variable of interest | 5 |
| Outcome data | 15* | Report numbers of outcome events or summary measures | 7-8 |
| Main results | 16 | (*a*) Give unadjusted estimates and, if applicable, confounder-adjusted estimates and their precision (eg, 95% confidence interval). Make clear which confounders were adjusted for and why they were included | 7-9 |
|  |  | (*b*) Report category boundaries when continuous variables were categorized | 7 |
|  |  | (*c*) If relevant, consider translating estimates of relative risk into absolute risk for a meaningful time period | NA |
| Other analyses | 17 | Report other analyses done—eg analyses of subgroups and interactions, and sensitivity analyses | 7-9, appendix |
| Discussion | | | |
| Key results | 18 | Summarise key results with reference to study objectives | 9-12 |
| Limitations | 19 | Discuss limitations of the study, taking into account sources of potential bias or imprecision. Discuss both direction and magnitude of any potential bias | 12-13 |
| Interpretation | 20 | Give a cautious overall interpretation of results considering objectives, limitations, multiplicity of analyses, results from similar studies, and other relevant evidence | 13 |
| Generalisability | 21 | Discuss the generalisability (external validity) of the study results | 9-13 |
| Other information | | | |
| Funding | 22 | Give the source of funding and the role of the funders for the present study and, if applicable, for the original study on which the present article is based |  |

*Give information separately for exposed and unexposed groups.

**Note:** An Explanation and Elaboration article discusses each checklist item and gives methodological background and published examples of transparent reporting. The STROBE checklist is best used in conjunction with this article (freely available on the Web sites of PLoS Medicine at http://www.plosmedicine.org/, Annals of Internal Medicine at http://www.annals.org/, and Epidemiology at http://www.epidem.com/). Information on the STROBE Initiative is available at [www.strobe-statement.org](http://www.strobe-statement.org).

**S2. Sample size calculation**

We used sample size calculation formula for cross-sectional studies to estimate a prevalence [1]. Based on our pilot study among 786 children parent, a total of 4009 number of girls’ parent are needed with expected 12.7% prevalence, 95% confidence interval, precision of 10%, response rate of 70%.

**S3. Precaution Adoption Process Model modification**

The original Precaution Adoption Process Model (PAPM) divided the preventive behavior into seven categorizes including unaware, unengaged, deciding about acting, deciding to act, acting, decided not to act, and maintenance. We have amended the PAPM to better test parental HPV vaccination decisions for their girls. Previous study using PAPM to measure parental HPV vaccination decision reported 0.9% of parents decided not to vaccinate their girls. The research team decided to change the stage of “decided not to act” to “not to vaccinate so far”, thus to better grasp the momentous parental intention. Moreover, the study did not aim to test the factors influencing parental HPV vaccination refusal. HPV vaccination behavior is a one-time behavior in our study, thus we deleted the options of “unengaged” and “maintenance” and merged the option of “deciding about acting” and “deciding to act” into one category named “decided to vaccinate”. The stage of “acting” was changed as “vaccinated” in this study. Consequently, the modified model classified parental vaccination decision into six stages including unengaged in child HPV vaccination decision, unaware, undecided, not to vaccinate so far, decided to vaccinate, and vaccinated. Parents who reported unengaged in child HPV vaccination decision were excluded for analysis.

**S4. Sensitive analysis**

Sensitive analysis was conducted to confirm the robotic of the study results. In the original model, parents’ whose girls have already been vaccinated were categorized into parents who intended to get their girls vaccinated. We exclude the 257 parents who reported the behaviour before investigation. These sensitivity analyses support the main findings.

Sensitive analysis

|  | Intended to get vaccinated n=3870  (exclude already vaccinated) | Intend to choose HPV-9 and HPV-4 |
| --- | --- | --- |
|  | aOR (95%CI) | aOR (95%CI) |
| Intercept | 0.12(0.05,0.27)*** | 0.17(0.05,0.59)** |
| Girls’ age | 1.05(0.99,1.12) | 1.06(0.98,1.15) |
| Girls’ ethnicity |  |  |
| Han | Ref | Ref |
| Minority | 0.80(0.63,1.01) | 0.96(0.61,1.51) |
| Province |  |  |
| Zhejiang | Ref | Ref |
| Guizhou | 0.69(0.52,0.91)** | 0.65(0.48,0.89)** |
| Urbanization |  |  |
| Urban | Ref | Ref |
| Rural | 0.87(0.66,1.15) | 0.70(052,0.94)* |
| Parents’ education |  |  |
| Primary and under | Ref | Ref |
| Middle school | 1.14(0.88,1.50) | 1.10(0.68,1.77) |
| High school | 1.32(0.99,1.78) | 1.57(0.94,2.64) |
| College and above | 1.50(1.10,2.06)* | 3.35(1.91,5.87)*** |
| Household Income (monthly) |  |  |
| <=3000 | Ref | Ref |
| 3001-5000 | 1.31(1.06,1.62)* | 0.98(0.67,1.42) |
| 5001-10000 | 1.20(0.95,1.52) | 1.86(1.23,2.83)** |
| >10000 | 1.58(1.22,2.04)** | 2.22(1.42,3.48)** |
| Relationship |  |  |
| Father | Ref | Ref |
| Mother | 1.35(1.12,1.63)** | 2.68(1.90,3.78)*** |
| Mother received HPV vaccine^a^ |  |  |
| No/Don't know | Ref | Ref |
| Yes | 3.12(2.51,3.87)*** | 1.70(1.20,2.41)** |
| Mother screened for cervical cancer |  |  |
| No/Don't know | Ref | Ref |
| Yes | 1.82(1.56,2.12)*** | 1.99(1.51,2.63)*** |
| Parental health literacy |  |  |
| 1 | Ref | Ref |
| 2 | 0.92(0.77,1.10) | 0.93(0.67,1.29) |
| 3 | 1.25(0.96,1.64) | 1.32(0.81,2.16) |
| 4 | 1.19(0.97,1.45) | 1.01(0.71,1.44) |

1. Naing L, Nordin RB, Abdul Rahman H, Naing YT. Sample size calculation for prevalence studies using Scalex and ScalaR calculators. BMC Medical Research Methodology **2022**; 22(1): 209.
